# Postoperative residual patellar bone thickness and patellar height in patients with quadriceps tendon rupture after total knee arthroplasty: A retrospective case‐control study

**DOI:** 10.1002/jeo2.70966

**Published:** 2026-10-29

**Authors:** Davide Pellicanò, Mirco Lo Presti, Maria Pia Neri, Andrea Maria Di Prima, Salvatore Rubino, Stefano Zaffagnini

**Affiliations:** ^1^ II Orthopaedic and Traumatology Clinic, IRCCS Istituto Ortopedico Rizzoli Bologna Italy

**Keywords:** extensor mechanism failure, patellar height, patellar thickness, quadriceps tendon rupture, total knee arthroplasty

## Abstract

**Purpose:**

Quadriceps tendon rupture following total knee arthroplasty is a rare but serious complication. This study aimed to compare postoperative residual patellar bone thickness and patellar height between patients who developed quadriceps tendon rupture after primary total knee arthroplasty and matched controls.

**Methods:**

A retrospective case‐control study included 17 patients who developed quadriceps tendon rupture within 6 months after primary total knee arthroplasty and 100 controls without extensor mechanism complications. Controls were selected using group‐level frequency matching for age, sex and body mass index. All patients underwent patellar resurfacing. Patellar height was assessed preoperatively and postoperatively using the Insall–Salvati index, while postoperative residual patellar bone thickness was measured on immediate postoperative lateral radiographs. Group comparisons were performed using the Wilcoxon rank‐sum and Fisher's exact tests. Logistic regression was used to evaluate the association between residual patellar bone thickness and tendon rupture, with adjustment for age, sex and body mass index.

**Results:**

No significant differences were observed between groups in age, sex, body mass index or diabetes mellitus. Median postoperative residual patellar bone thickness was numerically lower in patients with tendon rupture than in controls (11.3 vs. 11.8 mm), but the between‐group difference was not statistically significant. Residual patellar bone thickness was also not significantly associated with tendon rupture after adjustment for age, sex and body mass index (adjusted odds ratio 0.77, 95% confidence interval 0.55–1.08; *p* = 0.127). No significant differences were observed in preoperative or postoperative Insall‐Salvati index or changes in patellar height.

**Conclusions:**

No significant association was identified between postoperative residual patellar bone thickness, patellar height parameters and quadriceps tendon rupture in this cohort. Given the limited number of rupture cases, smaller or moderate associations cannot be excluded.

**Level of Evidence:**

Level III.

**Abbreviations**
BMI: body mass index
CI: confidence interval
DM: diabetes mellitus
IQR: interquartile range
I‐S: Insall–Salvati index
OR: odds ratio
SD: standard deviation
TKA: total knee arthroplasty

## INTRODUCTION

Total knee arthroplasty (TKA) is one of the most effective and commonly performed surgical procedures for the treatment of advanced knee osteoarthritis, providing substantial pain relief and functional improvement in the majority of patients [2, 12]. Despite the excellent clinical outcomes and the increasing number of procedures performed worldwide, postoperative complications may still occur, including infection, instability, stiffness and mechanical complications involving the extensor mechanism [2, 11, 12].

Among these, quadriceps tendon rupture represents a rare but highly debilitating complication, often associated with loss of active knee extension, functional impairment, pain and deterioration in quality of life [11, 13, 16, 17, 18]. The incidence reported in the literature ranges from 0.1% to 2.5% following TKA [2, 10, 13]. Furthermore, surgical treatment of these injuries remains challenging and is frequently associated with suboptimal clinical outcomes, high complication rates and failure of extensor mechanism reconstruction [1, 3, 7, 11, 13, 15, 16, 17, 18].

The etiology of extensor mechanism disruption following TKA is considered multifactorial [2, 5, 11]. Several predisposing factors have been described in the literature, including systemic comorbidities such as diabetes mellitus, obesity and chronic corticosteroid therapy [10], surgical factors such as component malrotation, lateral retinacular release and joint line elevation [2, 8, 11], as well as local factors including tendon degeneration, previous extensor mechanism injury and vascular compromise of the tendon [2, 8].

In recent years, increasing attention has been directed towards the role of patellofemoral biomechanics after TKA [4, 6, 9, 14]. Alterations in patellar height and patellar thickness may modify the extensor mechanism lever arm and the forces acting across the patellofemoral joint, potentially influencing the mechanical load applied to the quadriceps tendon [6, 8, 14]. In resurfaced patellae, both composite patellar thickness and the amount of residual patellar bone may be relevant to postoperative patellofemoral biomechanics [4, 6, 9].

However, although several studies have investigated the influence of patellar parameters on clinical outcomes and patellofemoral complications after TKA, their potential role in the risk of quadriceps tendon rupture remains poorly investigated [4, 5, 6, 9, 14].

Therefore, the aim of the present study was to evaluate the radiographic characteristics of patients who developed quadriceps tendon rupture following TKA compared with a control group, with particular attention to postoperative residual patellar bone thickness after resurfacing and pre and postoperative patellar height.

## MATERIALS AND METHODS

### Study design and patient selection

A retrospective case‐control study was conducted at our Institution to investigate the association between radiographic patellar parameters and quadriceps tendon rupture following primary TKA. Patients who underwent primary TKA between January 2018 and February 2025 were screened for eligibility. The case group consisted of patients who developed quadriceps tendon rupture within 6 months after primary TKA. Diagnosis was established on the basis of clinical examination and/or ultrasonographic assessment, according to the available clinical documentation. Clinical findings included impairment of active knee extension and, when documented, a palpable tendon defect.

Patients were eligible for the case group if they were aged 18 years or older, had undergone primary TKA during the study period, had developed quadriceps tendon rupture within 6 months after surgery, and had adequate preoperative and immediate postoperative lateral radiographs together with sufficient clinical documentation. Exclusion criteria were revision TKA, previous quadriceps tendon injury or repair, severe concomitant orthopaedic or systemic conditions potentially affecting the extensor mechanism, periprosthetic joint infection requiring implant explantation and incomplete radiographic or clinical data. Eighteen patients with quadriceps tendon rupture were initially identified; one patient was excluded because of periprosthetic joint infection requiring implant explantation, leaving 17 patients in the final case group.

The control group consisted of 100 patients who underwent primary TKA during the same study period and did not develop quadriceps tendon rupture or other extensor mechanism complications during a minimum follow‐up of 6 months. Controls were required to be aged 18 years or older and to have adequate preoperative and immediate postoperative lateral radiographs together with sufficient clinical documentation; the same exclusion criteria applied as for the case group. Controls were selected to achieve an overall distribution of age, sex and body mass index (BMI) comparable to that of the case group. This selection was guided by the distribution of these variables among cases, using approximate rather than rigid matching ranges and eligible controls were then randomly selected. No missing values were present for the variables included in the final analytical dataset, and no data imputation was performed.

Relevant systemic comorbidities potentially affecting tendon quality, including renal disease, history of renal transplantation and chronic corticosteroid exposure, were also reviewed from the available medical records when documented. The patient selection process is summarised in Figure 1.

**Figure 1:**
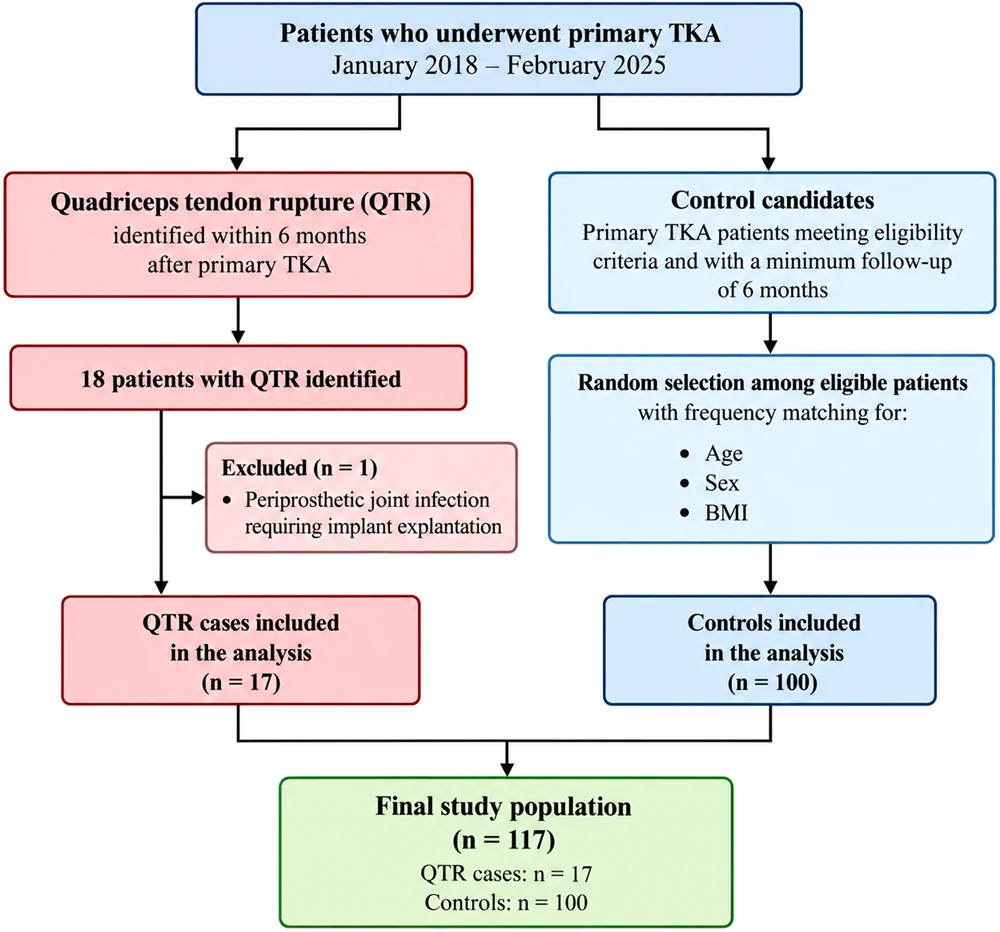
Flow diagram of patient selection and study‐group allocation. BMI, body mass index; QTR, quadriceps tendon rupture; TKA, total knee arthroplasty.

### Surgical technique

Primary TKAs were performed by three surgeons with comparable experience, using a standardised operative technique. A medial parapatellar approach was used in all cases. Patellar resurfacing was routinely performed in all patients, and no lateral retinacular release was carried out. The operative technique and patellar management strategy remained unchanged throughout the study period (2018–2025).

### Radiographic assessment

Radiographic evaluation was performed using standardised preoperative and immediate postoperative lateral knee radiographs. Immediate postoperative lateral radiographs were obtained with the patient in the supine position and the knee in approximately 0°–30° of flexion. Radiographic positioning was adjusted to achieve superimposition of the femoral condyles and a true lateral projection of the tibial plateau as closely as possible.

All primary radiographic measurements were performed using the institutional Vue picture archiving and communication system by a single observer following a standardised measurement protocol. The primary observer was aware of case/control status. Interobserver reliability was assessed in a randomly selected subset corresponding to one‐third of the study cohort (*n* = 39 radiographs). Measurements in this subset were independently repeated by a second observer who was blinded to case/control status, and agreement was evaluated using a two‐way random‐effects, absolute‐agreement intraclass correlation coefficient [ICC(2,1)].

Patellar height was assessed preoperatively and postoperatively using the Insall‐Salvati index (I‐S). The I‐S was selected because of its widespread clinical use, reproducibility and applicability on standard lateral radiographs, while previous studies have demonstrated comparable reliability among commonly used patellar height measurement methods following TKA [9, 14]. As the I‐S is expressed as a ratio between patellar tendon length and patellar length, radiographic calibration was not required.

Patellar resurfacing was routinely performed during primary TKA in all patients included in the study. Therefore, the radiographic patellar thickness variable evaluated in the present study represented the residual patellar bone thickness after patellar resection, rather than native or composite patellar thickness. Resurfacing was performed in 100% of patients in both the quadriceps tendon rupture and control groups.

Postoperative residual patellar bone thickness was measured on immediate postoperative lateral radiographs. It was defined as the maximum distance, measured on the lateral radiographic projection, between the anterior cortical surface of the patella and the resected osseous surface of the articular side, corresponding to the supporting plane of the patellar component. The patellar component itself was not included in the measurement.

To account for radiographic magnification, postoperative radiographs were calibrated using a clearly identifiable prosthetic structure with a manufacturer‐specified dimension that remained constant across the available implant sizes. The reference structure was selected separately for each implant system on the basis of the corresponding technical documentation and was used consistently for all patients implanted with the same prosthetic design. Depending on the implant system, calibration references included fixed dimensions such as tibial baseplate thickness, tibial stem thickness or tibial stem length. This approach was adopted to minimise variability related to implant size and radiographic magnification. A representative example of the calibration and measurement technique is shown in Figure 2.

**Figure 2:**
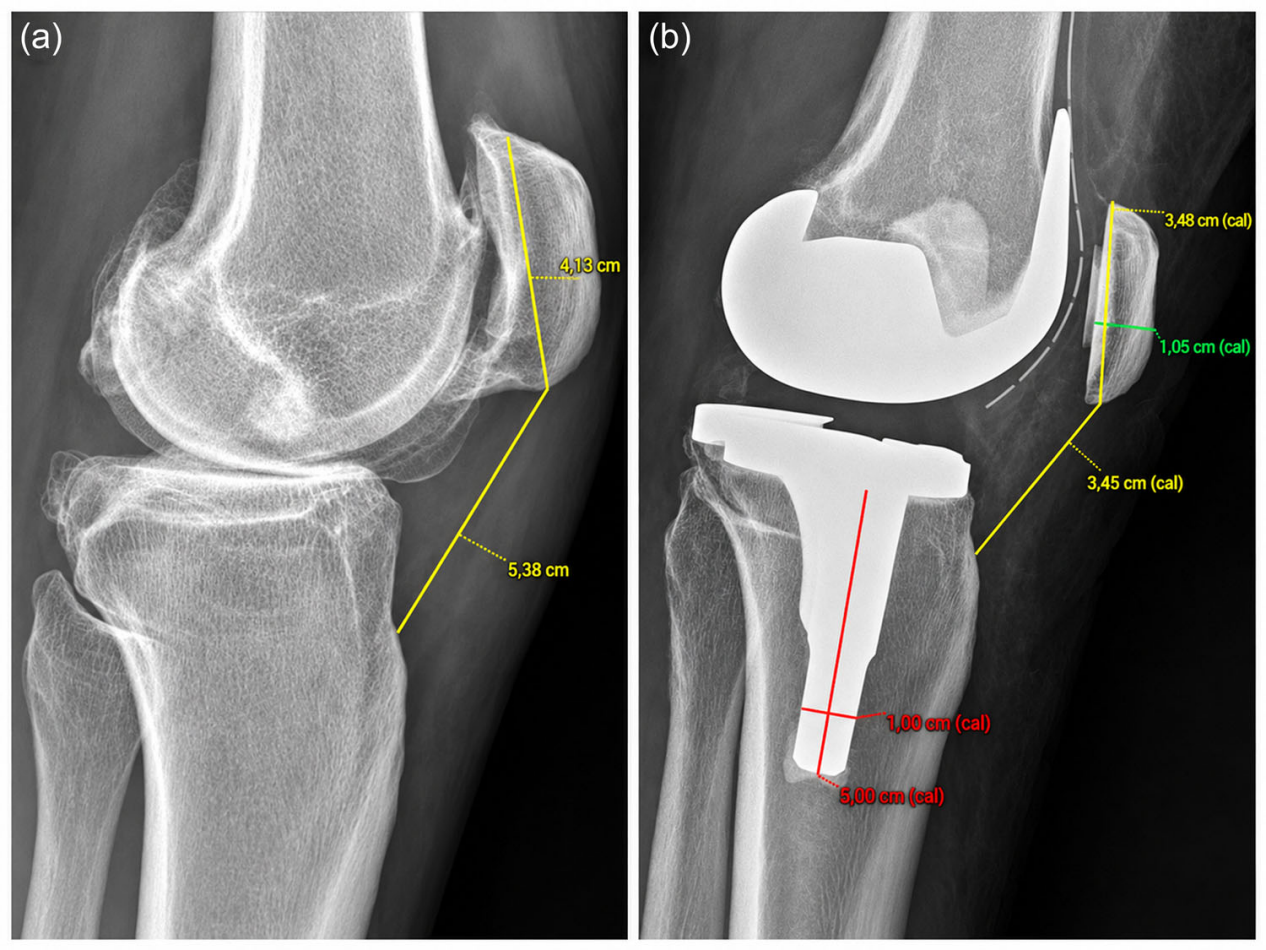
(a) Preoperative lateral knee radiograph showing the measurements used to calculate the Insall–Salvati index. (b) Immediate postoperative lateral radiograph showing postoperative Insall‐Salvati measurement, residual patellar bone thickness and radiographic calibration using prosthetic components of known dimensions.

The yellow lines labelled *X* indicate the measurements used to calculate the preoperative and postoperative Insall‐Salvati indices. The green line labelled *Y* indicates residual patellar bone thickness, while the red lines labelled *Z* show an example of radiographic calibration using prosthetic components of known dimensions.

### Data collection

Demographic, clinical, surgical and radiographic data were collected from institutional medical records.

The following variables were recorded: age at surgery, sex, BMI, diabetes mellitus status, implanted prosthetic model, preoperative I‐S, postoperative I‐S and postoperative residual patellar bone thickness. The change in Δ I‐S was calculated as the postoperative I‐S minus the preoperative I‐S (Δ I‐S = postoperative I‐S − preoperative I‐S).

For patients who developed quadriceps tendon rupture, additional clinical characteristics were collected, including time from primary TKA to rupture, traumatic or atraumatic mechanism, extent of the tear (complete or partial), anatomical location of the rupture, ultrasonographic assessment, presence of an active extension deficit and subsequent surgical treatment.

### Outcome measure

The primary outcome of the study was the occurrence of quadriceps tendon rupture within 6 months following primary TKA.

### Statistical analysis

Continuous variables were summarised as median, interquartile range (IQR), minimum, maximum, mean and standard deviation (SD). Categorical variables were reported as frequencies and percentages.

Comparisons between patients who developed quadriceps tendon rupture and the control group were performed using the Wilcoxon rank‐sum test for continuous variables and Fisher's exact test for categorical variables.

The association between postoperative residual patellar bone thickness and quadriceps tendon rupture was initially evaluated using unadjusted logistic regression. Given the frequency‐matched case‐control design, an unconditional multivariable logistic regression model was subsequently performed adjusting for the matching variables age, sex and BMI. Quadriceps tendon rupture was coded as the outcome event (QTR = 1; control = 0). Results were reported as odds ratios (ORs) with corresponding 95% confidence intervals (CIs).

A two‐sided *p*‐value < 0.05 was considered statistically significant.

All statistical analyses were performed using R statistical software (R Core Team, 2024; R Foundation for Statistical Computing).

## RESULTS

### Patient characteristics

Eighteen patients with quadriceps tendon rupture following primary TKA were initially identified. One patient was excluded because of a periprosthetic joint infection requiring implant explantation, leaving 17 patients in the final case group. A total of 100 eligible control patients with a minimum follow‐up of 6 months were randomly selected and frequency‐matched for age, sex and BMI. The patient‐selection process is shown in Figure 1.

A total of 117 patients were included in the study, comprising 17 patients who developed quadriceps tendon rupture within 6 months following primary TKA and 100 controls without extensor mechanism complications.

Baseline demographic and clinical characteristics are summarised in Table 1. No significant differences were observed between groups regarding age, sex distribution, BMI or diabetes mellitus status. The median age at the time of TKA was 72.0 years (IQR: 68.0–76.0) in the quadriceps tendon rupture group and 71.5 years (IQR: 66.0–76.0) in the control group (*p* = 0.745). Similarly, BMI was comparable between groups (27.9 ± 3.7 vs. 28.5 ± 4.3 kg/m^2^, *p* = 0.757). Diabetes mellitus was present in 17.6% of cases and 13.0% of controls (*p* = 0.701).

**Table 1:** Baseline demographic and clinical characteristics of patients with quadriceps tendon rupture and controls.

	Quadriceps tendon rupture group *N* = 17	Control group *N* = 100	*p*‐Value
Age at TKA (Years)			0.745
Median (Q1, Q3)	72.0 (68.0, 76.0)	71.5 (66.0, 76.0)	
(Min, Max)	(61.0, 84.0)	(51.0, 88.0)	
Mean (SD)	72.1 (6.7)	71.4 (7.1)	
Sex			0.759
Female	14 (82.4)	75 (75.0)	
Male	3 (17.6)	25 (25.0)	
BMI			0.757
Median (Q1, Q3)	28.3 (25.5, 30.1)	27.5 (25.3, 31.3)	
(Min, Max)	(21.5, 36.1)	(20.7, 40.1)	
Mean (SD)	27.9 (3.7)	28.5 (4.3)	
DM			0.701
No	14 (82.4)	87 (87.0)	
Yes	3 (17.6)	13 (13.0)	

Abbreviations: BMI, body mass index; DM, diabetes mellitus; SD, standard deviation; TKA, total knee arthroplasty.

Several prosthetic designs were represented in both groups. The most commonly implanted model was Attune DePuy PS, used in 11 of 17 cases (64.7%) and 64 of 100 controls (64.0%), followed by Physica Lima (11.8% and 11.0%, respectively). Other implant models were less frequently represented and included Journey II and Legion by Smith & Nephew, Zimmer Persona, TC3 DePuy, SymphoKnee LINK and Attune fixed‐bearing designs.

### Clinical characteristics of quadriceps tendon ruptures

Among the 17 patients with quadriceps tendon rupture, the median time from primary TKA to rupture was 60 days (IQR: 24–120; range 1–173). Eleven ruptures (64.7%) followed a documented traumatic mechanism, 5 (29.4%) were atraumatic and one occurred during physiotherapy with an unclear mechanism. Twelve tears (70.6%) were complete and 5 (29.4%) partial. Seven ruptures involved the patellar insertion, 7 the tendon substance and 3 the myotendinous junction. Ultrasonography was performed in 11 patients (64.7%), an active extension deficit was documented in 12 (70.6%) and 12 patients (70.6%) underwent surgical treatment.

### Postoperative residual patellar bone thickness

Residual patellar bone thickness measurements are reported in Table 2.

**Table 2:** Postoperative residual patellar bone thickness in patients with quadriceps tendon rupture and controls.

Residual patellar bone thickness (mm)	Quadriceps tendon rupture group *N* = 17	Control group *N* = 100	*p*‐Value (from Wilcoxon rank‐sum test)
Median (Q1, Q3)	11.3 (10.2, 12.4)	11.8 (10.8, 12.9)	0.296
(Min, Max)	(7.5, 14.2)	(8.3, 17.1)	
Mean (SD)	11.2 (2.0)	11.9 (1.7)	

Abbreviation: SD, standard deviation.

Interobserver reliability for residual patellar bone thickness was excellent, with an ICC of 0.988 (95% CI, 0.980–0.992).

Median residual patellar bone thickness was 11.3 mm (IQR: 10.2–12.4) in patients who developed quadriceps tendon rupture and 11.8 mm (IQR: 10.8–12.9) in controls.

In the unadjusted logistic regression analysis, postoperative residual patellar bone thickness was not significantly associated with quadriceps tendon rupture (OR: 0.77 per 1‐mm increase, 95% CI, 0.56–1.06, *p* = 0.103). After adjustment for the frequency‐matching variables age, sex and BMI, the association remained nonsignificant (adjusted OR: 0.77, 95% CI, 0.55–1.08, *p* = 0.127). The distribution of residual patellar bone thickness according to the study group is illustrated in Figure 3, while the unadjusted and adjusted logistic regression analyses are summarised in Table 3.

**Figure 3:**
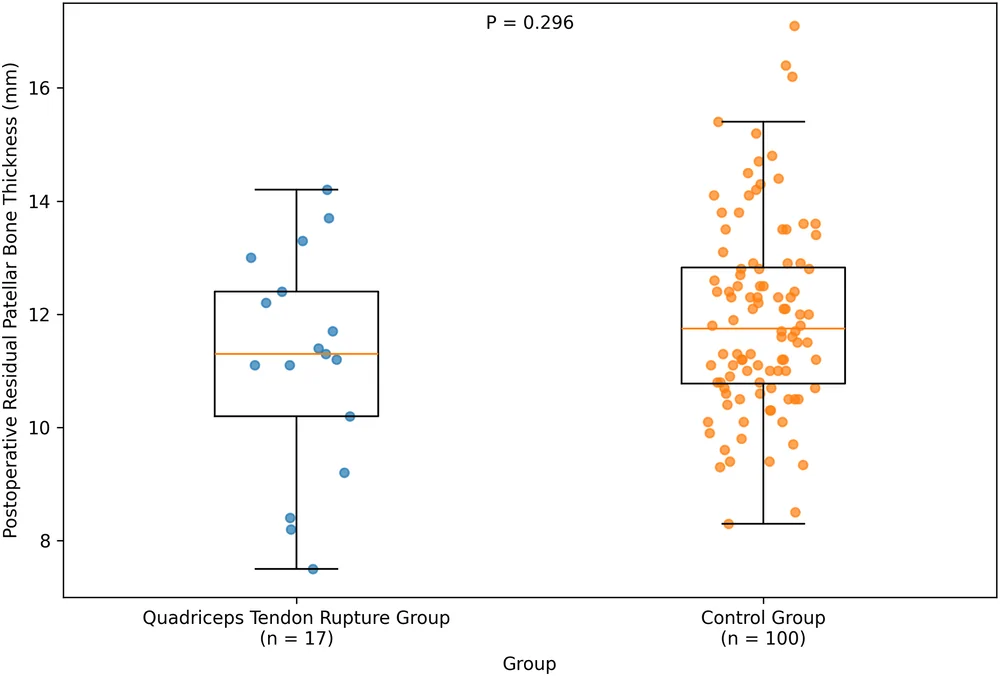
Distribution of postoperative residual patellar bone thickness in patients with quadriceps tendon rupture and controls.

**Table 3:** Logistic regression analysis of residual patellar bone thickness and quadriceps tendon rupture.

Model	OR	95% CI	*p*‐Value
Unadjusted			
Residual patellar bone thickness, per 1‐mm increase	0.77	0.56–1.06	0.103
Adjusted model^a^			
Residual patellar bone thickness, per 1‐mm increase	0.77	0.55–1.08	0.127
Age, per year	1.01	0.93–1.09	0.899
Male sex^b^	0.82	0.19–3.62	0.795
BMI, per kg/m^2^	0.97	0.85–1.10	0.615

Abbreviations: BMI, body mass index; CI, confidence interval; OR, odds ratio.

^a^ Multivariable logistic regression model adjusted for age, sex, and body mass index.

^b^ Female sex was used as the reference category for the sex variable.

### Patellar height assessment

Patellar height measurements based on the I‐S are summarised in Table 4.

**Table 4:** Preoperative, postoperative and change in Insall‐Salvati index by study group.

	Quadriceps tendon rupture group *N* = 17	Control group *N* = 100	*p*‐Value^a^
I‐S Pre			0.359
Median (Q1, Q3)	1.1 (1.1, 1.2)	1.2 (1.1, 1.3)	
(Min, Max)	(0.9, 1.5)	(0.9, 1.6)	
Mean (SD)	1.2 (0.2)	1.2 (0.2)	
I‐S Post			0.448
Median (Q1, Q3)	1.1 (1.0, 1.2)	1.1 (1.0, 1.2)	
(Min, Max)	(0.9, 1.4)	(0.8, 1.5)	
Mean (SD)	1.1 (0.1)	1.1 (0.1)	
Δ I‐S			0.662
Median (Q1, Q3)	−0.1 (−0.1, −0.1)	−0.1 (−0.2, 0.0)	
(Min, Max)	(−0.3, 0.2)	(−0.4, 0.2)	
Mean (SD)	−0.1 (0.1)	−0.1 (0.1)	

Abbreviation: SD, standard deviation.

^a^ Wilcoxon rank sum test.

No significant differences were observed between groups in preoperative I‐S values (*p* = 0.359). Similarly, postoperative I‐S values were comparable between patients with quadriceps tendon rupture and controls (*p* = 0.448).

The change in I‐S, calculated as postoperative minus preoperative I‐S (Δ I‐S), showed no statistically significant difference between groups (*p* = 0.662).

Overall, neither preoperative nor postoperative patellar height, nor changes in patellar height following TKA, were associated with the occurrence of quadriceps tendon rupture.

The distribution of Δ I‐S values in the two groups is shown in Figure 4.

**Figure 4:**
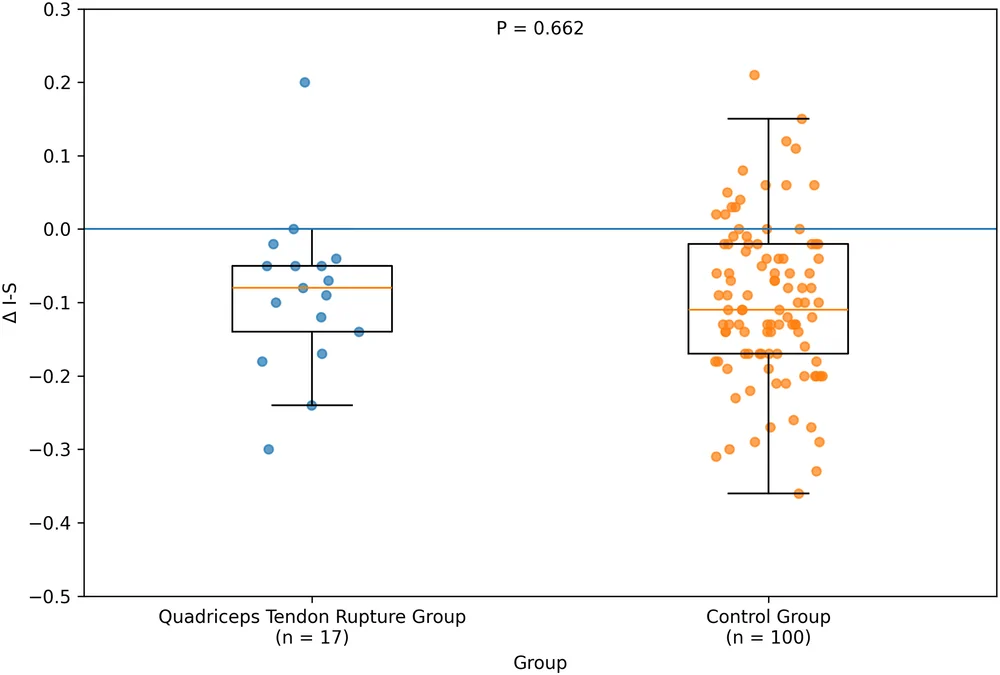
Distribution of Δ Insall–Salvati index, calculated as postoperative minus preoperative I‐S, in patients with quadriceps tendon rupture and controls.

## DISCUSSION

Quadriceps tendon rupture remains a rare but severe complication following primary TKA, frequently resulting in substantial functional impairment and requiring complex treatment. The present cohort also demonstrated considerable clinical heterogeneity: approximately two‐thirds of ruptures followed a documented traumatic mechanism, whereas nearly one‐third were atraumatic, and both complete and partial tears were observed at different anatomical levels. These findings support the concept that quadriceps tendon failure after TKA may arise through different mechanical and biological pathways rather than representing a single uniform clinical entity.

The main finding of the present study was that neither postoperative residual patellar bone thickness after resurfacing nor pre or postoperative patellar height differed significantly between patients who developed quadriceps tendon rupture and controls. Residual patellar bone thickness was also not significantly associated with rupture in either the unadjusted analysis or after adjustment for age, sex and BMI. Although patients with tendon rupture showed numerically lower residual patellar bone thickness, the CIs around the ORs included the null value; therefore, smaller or moderate associations cannot be excluded.

The rationale for investigating these parameters derives from the established influence of patellar morphology on patellofemoral biomechanics and postoperative knee function [6, 8, 9, 14]. In resurfaced patellae, a distinction should be made between composite patellar thickness, which includes the residual bone and prosthetic component, and residual patellar bone thickness after resection; the present study specifically evaluated the latter. Although patellar thickness and height may affect extensor mechanism biomechanics, our findings suggest that these static radiographic parameters alone are unlikely to explain quadriceps tendon rupture.

This interpretation is consistent with the multifactorial nature of extensor mechanism failure described in the literature [2, 10, 11, 13]. Patient‐related factors, tendon biology, surgical technique and postoperative mechanical stresses may all contribute to tendon vulnerability. Systemic conditions such as diabetes, obesity, renal or metabolic disease, chronic corticosteroid exposure and degenerative tendon changes may interact with surgical factors, including soft‐tissue handling, vascular compromise, component positioning and extensor mechanism tension. Moreover, conventional radiographs provide only a static representation of a dynamic biomechanical system and cannot characterise the forces transmitted through the quadriceps tendon during functional activities or traumatic events.

The present study has several strengths. It represents a relatively large single‐centre case‐control series specifically investigating radiographic patellar parameters in patients with quadriceps tendon rupture after primary TKA. Controls were randomly selected and frequency‐matched for age, sex and BMI, and all radiographic measurements were obtained from immediate postoperative images using a standardised protocol. This allowed evaluation of the anatomical conditions created at surgery before subsequent remodelling or changes in patellar position.

Several limitations should nevertheless be acknowledged. First, the retrospective design introduces the possibility of selection, information and residual confounding bias. Some systemic factors potentially affecting tendon quality, including chronic kidney disease, dialysis status, secondary hyperparathyroidism and chronic corticosteroid exposure, were not systematically recorded in all patients and could not be comprehensively analysed. Second, although the primary radiographic observer was not blinded to case/control status, interobserver reproducibility assessed by a second blinded observer in a randomly selected subset was excellent for residual patellar bone thickness. Third, the limited number of rupture cases, reflecting the rarity of this complication, reduced the precision of the regression estimates, as indicated by the CIs around the ORs.

Additional methodological limitations concern the radiographic measurements. Preoperative patellar thickness and the corresponding change in thickness could not be reliably assessed because preoperative radiographs lacked a reference of known dimensions for calibration; therefore, postoperative residual bone thickness cannot distinguish between native patellar morphology and the amount of bone resected during resurfacing. Although postoperative calibration was standardised using manufacturer‐specified prosthetic dimensions that remained constant across implant sizes, different reference structures were required for different implant systems and the calibration approach has not been formally validated against an external reference standard. Furthermore, the study evaluated residual rather than composite patellar thickness. Finally, patellar height was assessed exclusively using the I‐S, which does not directly account for the relationship between the patella and the postoperative joint line; consequently, pseudo‐patella baja related to joint‐line elevation may not have been fully captured.

## CONCLUSION AND FUTURE RESEARCH

In this retrospective case‐control study, no significant association was identified between postoperative residual patellar bone thickness after resurfacing, pre and postoperative patellar height or changes in patellar height and quadriceps tendon rupture following primary TKA. These findings should be interpreted in light of the limited number of rupture cases and the resulting uncertainty around the effect estimates. Further multicenter investigations with larger cohorts are needed to better define the relative contribution of biomechanical, surgical and patient‐related factors to quadriceps tendon rupture after TKA. Future prospective studies incorporating dedicated preoperative assessment of quadriceps tendon morphology and structural quality, ideally by ultrasonography or MRI, may also help clarify the potential role of intrinsic tendon vulnerability and preexisting degeneration in the development of postoperative rupture.

## AUTHOR CONTRIBUTIONS

**Davide Pellicanò**: Conceptualisation; methodology; investigation; data curation; formal analysis; writing—original draft. **Mirco Lo Presti**: Interpretation of results; writing—review and editing. **Maria Pia Neri**: Interpretation of results; writing—review and editing. **Andrea Maria Di Prima**: Methodology; investigation; data curation; formal analysis. **Salvatore Rubino**: Investigation; data curation. **Stefano Zaffagnini**: Conceptualisation; methodology; supervision; writing—review and editing.

## FUNDING INFORMATION

The authors have no funding to report.

### DECLARATION OF GENERATIVE AI AND AI‐ASSISTED TECHNOLOGIES IN THE MANUSCRIPT PREPARATION PROCESS

During the preparation of this work, the author(s) used ChatGPT (OpenAI, San Francisco, CA, USA) exclusively for language editing and refinement of academic English. No artificial intelligence tools were used for data analysis, interpretation of results or generation of scientific conclusions. The author(s) reviewed and edited the output as needed and take full responsibility for the content of the published article.

## CONFLICT OF INTEREST STATEMENT

The authors declare no conflicts of interest.

## ETHICS STATEMENT

The study was approved by the Comitato Etico Area Vasta Emilia Centro (CE‐AVEC), approval reference CE‐AVEC 210/2025/Oss/IOR. Written informed consent was obtained from all participants included in the study.

## Data Availability

The data that support the findings of this study are available from the corresponding author upon reasonable request.
